# Mental representation of climate-relevant behaviours: Confirmatory testing of similarity patterns obtained in a card sorting task by young adults

**DOI:** 10.3389/fpsyg.2023.1117452

**Published:** 2023-02-09

**Authors:** Sebastian Seebauer, Hans Peter Ellmer

**Affiliations:** LIFE Institute for Climate, Energy and Society, Joanneum Research Forschungsgesellschaft mbH, Graz, Austria

**Keywords:** shared concept, pro-environmental behaviour, grouping, spillover mechanism, category system, taxonomy, mental model

## Abstract

Efforts to promote climate-friendly consumption need to address groups of interrelated behaviours; however, experts and laypeople have different perspectives on which climate-relevant behaviours belong together. Understanding laypeople’s mental representations, or the perceived similarity of behaviours, may provide orientation on which behaviours should be promoted in concert in order to communicate comprehensibly and to catalyse spillover. The present study uses data on perceived similarity between 22 climate-relevant behaviours collected from 413 young adults in Austria in an open card sorting task. Five posited categorisations by domain, location, impact, difficulty, and frequency are tested in a confirmatory approach for their fit with the observed similarity patterns. By analysing co-occurrence matrices, edit distances and similarity indices, the best fit is found for the null hypothesis of random assignment. Ranking by test statistics shows that the domain categorisation fits next best, followed by impact, frequency, difficulty, and location. The categories of waste and advocacy behaviours emerge consistently in lay mental representations. The categories of behaviours with a high carbon footprint and difficult behaviours that are performed by few other people stand out from other, less extreme behaviours. Categorisation fit is not moderated by personal norms, stated competencies, and environmental knowledge. The analytical approaches for confirmatory testing of expected categorisations against observed similarity patterns may be applied to analyse any card sorting data.

## 1. Introduction

The grand challenge of combating climate change requires decarbonisation across all sectors, including the choices of private consumers (European Commission, 2019). As the achieved reductions in carbon emissions continue to fall below climate targets (Boehm et al., 2022), it becomes clear that focussing on selected consumer behaviours is no longer sufficient, but that changes across all aspects of private consumption are necessary (International Energy Agency, 2022). Thus, efforts at promoting climate-friendly consumption need to address groups of interrelated behaviours that can be tackled in concert.

Experts and laypeople, however, or those who design climate policy and those who are supposed to react to this policy in their daily lives, have a different understanding of which behaviours belong together. From the perspective of experts, all climate-relevant behaviours are conceptually linked in that they all contribute to a person’s carbon footprint. By contrast, laypeople hardly hold an overarching mental concept that carbon- and energy-intensive activities belong together (Truelove and Gillis, 2018). Instead, laypeople form their mental representations by grouping behaviours that relate to the same practices and habits, that are performed with the same domestic appliances or that hold the same meaning for themselves or a meaning shared with others (Gabe-Thomas et al., 2016; Doran et al., 2018).

Thus, experts need to promote multiple interrelated climate-friendly behaviours not (only) in a way that makes sense from a science or policy standpoint, but in a way that aligns with how their lay audience considers behaviours to be connected to each other. Adjusting to lay perceptions may render experts’ messages more comprehensible and actionable (see Section “1.1. Communicating comprehensibly by means of perceived similarity” below) and may leverage off existing behaviours to catalyse subsequent behaviour change in similar domains (Section “1.2. Catalysing spillover by means of perceived similarity”).

Laypeople’s mental representations of climate-relevant behaviours manifest, *inter alia*, in how they perceive some behaviours as similar and other behaviours as dissimilar. Consumers form mental representations through a cognitive process of grouping related objects, products or services according to their personal goals; this process results in their personal taxonomy of categories that constitute similarity (Loken, 2006; Gabe-Thomas et al., 2016). Sorting is an established technique for eliciting these taxonomies. Tasking people with ordering or grouping items allows identification of the criteria they use when categorising concepts by their similarities and differences (Gabe-Thomas et al., 2016; Doran et al., 2018). Sorting tasks may even reveal intuitive, implicit or unconscious categorisations. Thus, the present paper employs the sorting method to investigate which categorisations are key to the perceived similarity of various climate-relevant behaviours.

### 1.1. Communicating comprehensibly by means of perceived similarity

A clear understanding of perceived similarity is important for designing green marketing programmes that aim to encourage comprehensive changes spanning several climate-relevant behaviours (Thogersen, 2004). Programmes can be expected to be more successful if they cater to laypeople’s nuanced perceptions (Truelove and Gillis, 2018). Perceived similarity indicates how consumers arrange behaviours in their mental space and therefore shows entry points for promoting multiple climate-friendly behaviours together (Bernard et al., 2009; Kneebone et al., 2018).

Persuasive messages for broad lifestyle change are more comprehensible to consumers if they address those behaviours jointly which have the same meaning to the audience (Gabe-Thomas et al., 2016). Communication programmes should focus on themes and comparisons that link several perceptually similar behaviours (Bernard et al., 2009). Understanding where lay and expert taxonomies diverge allows the taking of dedicated steps to correct lay misperceptions (Doran et al., 2018).

### 1.2. Catalysing spillover by means of perceived similarity

Perceived similarity is prominently discussed to facilitate spillover, that is, one behaviour change triggering another behaviour change (Maki et al., 2019). Consumers transfer behavioural practices from one context to another because of their psychological need to maintain a self-image of being consistent and to avoid cognitive dissonance from being inconsistent (Thogersen, 2004). If a consumer does not see two behaviours as similar, performing one behaviour but not the other would not evoke feelings of inconsistency or dissonance. Thus, perceived similarity may be considered a precondition for spillover. Consumers may transfer behaviours between contexts they themselves see as similar, even if experts would regard these behaviours as disjoint and unrelated.

There is (still) considerable disagreement in the spillover literature on what makes behaviours similar: behaviours connected to the same motivational goal (Truelove et al., 2014; Nash et al., 2017), behaviours performed in contexts that are temporally or spatially close to each other (Thogersen and Crompton, 2009), or behaviours requiring similar effort, resources, and skills (Thogersen and Crompton, 2009; Margetts and Kashima, 2017; Höchli et al., 2019). The classification of behaviours as similar, and therefore prone to spillover, is mostly based on expert judgements; however, the perceptions of similarity by those consumers who engage in these behaviours are a better indicator of whether spillover might take place (Truelove et al., 2014; Maki et al., 2019). Thus, effective behavioural change programmes “should select key actions perceived as similar to, and thus able to be catalysed by, householders’ existing behaviours” (Kneebone et al., 2018:8).

### 1.3. Categorisations of climate-relevant behaviour

Categorisations assign behaviours to the same category if they are conceptually related. Previous research points to five categorisations that guide why laypeople group some behaviours as similar and distinguish other behaviours as dissimilar (Boudet et al., 2016): Domain, Location, Impact, Difficulty, and Frequency. The present study compares how these five categorisations appear in observed similarity patterns; next, these five categorisations are introduced in detail.

Similarity by consumption domain is arguably the most established categorisation (Kaiser and Wilson, 2004; Barr et al., 2005; DEFRA, 2008; Bernard et al., 2009; Blanken et al., 2015; Truelove and Gillis, 2018). In many studies, the domains of recycling and waste avoidance (e.g., separating waste, using returnable bottles), transport (e.g., using the car or public transport), and advocacy (e.g., environmental activism, participating in the public discourse) appear as distinct domains. However, there is less agreement on how to group domestic energy use for heating, hot water and electricity; nutrition choices; and shopping decisions for electronic devices, clothing and other consumer goods. Doran et al. (2018) report a catch-all category of energy use at home comprising energy-saving and efficient home appliances. Gabe-Thomas et al. (2016) report an everything-else cluster comprising water use, lighting, heating, and smaller electric devices. The present paper operationalises the *Domain* categorisation with the four categories energy use and consumption, transport, waste, and advocacy: the latter three categories are well established as distinct domains by previous research; energy use and consumption is adopted as an umbrella category for domestic and shopping behaviours.

Similarity by location refers to the places where behaviours are performed. The clustering of electrical appliances in Gabe-Thomas et al. (2016) differentiates between the locations kitchen (e.g., fridge, oven, kettle, dishwasher, washing machine, and tumble dryer) and entertainment (e.g., TV, computer, games console, and stereo system). Kneebone et al. (2018) distinguish indoor (kitchen, bathroom, and laundry) versus outdoor (garden) water use. Stern (2000) draws a general line between private versus public sphere environmentalism. Thus, the present paper operationalises the *Location* categorisation with the four categories indoor, outdoor, online, and political space, thereby differentiating whether behaviours are performed physically inside or outside the home, virtually in online commerce or on social media, or in the environment of the political debate.

Similarity by impact refers to the energy demand and related carbon emissions of behaviours. Truelove and Gillis (2018) propose environmental impact as a dimension of similarity. Energy is, however, an intangible and abstract concept to most laypeople: laypeople do not group items of similar energy demand unless directly asked to do so (Baird and Brier, 1981); laypeople do not understand energy consumption very well (Darby, 2006; Bartiaux, 2008) and “do not hold consistent mental models of energy as a concept” (Gabe-Thomas et al., 2016:11). Laypeople underrate the substantial, albeit indirect environmental impact of political behaviours (Truelove and Gillis, 2018). Still, as a dimension deemed critical by experts, the present paper operationalises the *Impact* categorisation *via* the relative shares of specific behaviours in the average personal carbon footprint with the four categories >10, 5−10, <5% and no direct impact. Carbon impacts are calculated by applying the ECHOES methodology to the study population (Bird et al., 2019). For instance, carbon emissions from using the car amount on average to 6.6% of all personal emissions; therefore, this behaviour is assigned to the 5−10% category. Advocacy behaviours are assigned to the no direct impact category, because they influence climate policy and may indirectly lead to a reduction in carbon emissions but do not directly affect the footprint of the person who engages in advocacy behaviours.

Similarity by difficulty refers to the effort in inconvenience, discomfort, time, and money required to perform a behaviour (Truelove and Gillis, 2018). Financial and behavioural cost, cognitive effort, and efficacy beliefs are repeatedly discussed as a dimension of similarity (Thogersen, 2004; Karlin et al., 2014; Boudet et al., 2016; Kneebone et al., 2018). The present paper operationalises the *Difficulty* categorisation with the four categories of 0−25, 26−50, 51−75, and 76−100% engagement probability. The higher the engagement probability, the less difficult the behaviour, as a higher percentage of the population is likely to engage in that very behaviour. Engagement probabilities are derived from a General Ecological Behaviour attitude distribution, using the selected behaviours reported in Kaiser and Wilson (2004), Kaiser et al. (2007), Kaiser et al. (2008), Kaiser and Schultz (2009), and Kaiser et al. (2010) for Swiss, Dutch, and German samples as signposts and mapping other behaviours by interpolation and analogy.

Finally, similarity by frequency refers to how often and regularly a behaviour is performed. Frequency is considered relevant in expert analyses of similarity (Karlin et al., 2014; Höchli et al., 2019). Behavioural frequency may overlap with behavioural difficulty: Laypeople do not differentiate frequency of action and financial cost (Truelove and Gillis, 2018). However, the Difficulty categorisation is based on how many people perform the behaviour, whereas the Frequency categorisation is based on how often people perform the behaviour. The present paper operationalises the *Frequency* categorisation with four factors as categories that are derived from a principal component analysis of self-reported behavioural frequency (see Section “2.3. Analytical approach”).

Most people seem to hold the same mental representations of perceived similarity. Doran et al. (2018) find that categorisations in a Norwegian and a German sample correspond highly. In Gabe-Thomas et al. (2016), the same cluster solution holds for female and male participants. By contrast, Thogersen (2004) finds that the moral importance of behaving environmentally responsibly moderates perceived similarity between behaviours. Thus, in the light of ambiguous previous research, the present paper explores moderator variables that could explain why people with a specific background or beliefs prefer some categorisations to others. Three potential moderator variables are analysed: One is personal norms (in other words, feeling morally obliged to engage in climate protection), because personal norms are broadly confirmed as a central factor in the cognitive processes influencing pro-environmental behaviour (Stern, 2000; Bamberg and Möser, 2007). The other two are environmental knowledge and stated competencies because informed and skilled people might be able to assess behaviours more precisely in the Impact and Difficulty categorisations (Frick et al., 2004; Steg and Vlek, 2009).

### 1.4. Aim of the paper

As argued above, perceived similarity is an important lever for communicating comprehensibly and for catalysing spillover. However, it is still unclear by which categorisations laypeople structure their mental representations of climate-relevant behaviours. The present paper extends previous exploratory research by carrying out confirmatory testing on how well the posited categorisations Domain, Location, Impact, Difficulty, and Frequency fit with observed similarity patterns. Perceived similarity is elicited in an open sorting task of climate-relevant behaviours in a sample of 413 young adults. In total, 22 behaviours (listed in Table 1) are analysed in order to cover a broad range of the climate-relevant actions private consumers may take (Truelove and Gillis, 2018). Personal norms, stated competencies, and environmental knowledge are tested for moderator effects on the fit of the posited categorisations.

**TABLE 1:** Assignment of behaviours to categories.

		Domain	Location	Impact	Difficulty	Frequency
1.	Heating	Energy use and consumption	Indoor	>10%	51−75%	Factor2
2.	Showering	Energy use and consumption	Indoor	<5%	76−100%	Factor4
3.	Saving hot water	Energy use and consumption	Indoor	<5%	76−100%	Factor2
4.	Turning on the light	Energy use and consumption	Indoor	<5%	76−100%	Factor2
5.	Using electronic devices	Energy use and consumption	Indoor	<5%	51−75%	Factor2
6.	Streaming video	Energy use and consumption	Online	<5%	51−75%	Factor4
7.	Separating waste	Waste	Indoor	<5%	76−100%	Factor4
8.	Avoiding plastic	Waste	Indoor	<5%	51−75%	Factor4
9.	Using the car	Transport	Outdoor	5−10%	26−50%	Factor3
10.	Using public transport	Transport	Outdoor	<5%	26−50%	Factor3
11.	Using the bicycle	Transport	Outdoor	<5%	51−75%	Factor3
12.	Flying	Transport	Outdoor	>10%	26−50%	Factor2
13.	Buying clothes	Energy use and consumption	Online	5−10%	26−50%	Factor2
14.	Buying electronic devices	Energy use and consumption	Online	<5%	51−75%	Factor2
15.	Eating meat	Energy use and consumption	Indoor	>10%	76−100%	Factor1
16.	Buying organic food	Energy use and consumption	Outdoor	5−10%	0−25%	Factor4
17.	Buying local food	Energy use and consumption	Outdoor	5−10%	26−50%	Factor4
18.	Participating in an NGO for climate protection	Advocacy	Political space	no direct impact	0−25%	Factor1
19.	Speaking out for climate protection online	Advocacy	Online	no direct impact	0−25%	Factor1
20.	Speaking with other people about climate protection	Advocacy	Outdoor	no direct impact	0−25%	Factor1
21.	Donating for climate protection	Advocacy	Online	no direct impact	0−25%	Factor1
22.	Demonstrating for climate protection	Advocacy	Political space	no direct impact	0−25%	Factor3

For criteria in assigning behaviours to posited categories, see section “1.3. Categorisations of climate-relevant behaviour”. NGO, non-governmental organisation.

The scope of the present paper does not include the categorisation of curtailment and efficiency behaviours, which is widely used in environmental psychology. Curtailment refers to actions that cut back consumption, whereas efficiency refers to actions that maintain consumption levels but require fewer carbon emissions or less energy (Gardner and Stern, 2008). Maintenance, that is, actions that require regular management, may constitute a third category but is close to efficiency (Karlin et al., 2014). The curtailment versus efficiency categorisation is not included in the analysis, because the analytical approach of the present paper requires a consistent number of four categories in all tested categorisations. Moreover, curtailment and efficiency overlap in some behaviours: for instance, car use (behaviour 9 in the present study, see Table 1) involves choosing the car type as well as everyday driving; or heating (behaviour 1) involves putting on a sweater and programming the thermostat as well as installing a non-fossil heating system. Boudet et al. (2016) and Doran et al. (2018) provide support for omitting this categorisation by showing that the notion of a curtailment versus efficiency dichotomy is not held by laypeople.

## 2. Materials and methods

### 2.1. Respondents

Standardised self-completion questionnaires were distributed from February to May 2020 to students in their final high school year (12th or 13th year of formal education), aged 17−21 years. The survey was implemented in 24 vocational and general secondary schools in urban and rural locations in the Austrian provinces of Styria and Tyrol. Students completed an online questionnaire in the classroom during school hours, using the school’s computers or their own electronic devices. A researcher was present on-site for oversight and clarification. Because of school closures in the COVID-19 national lockdown starting in mid-March 2020, however, data collection had to shift to an entirely online survey: teachers distributed an email invitation to the online survey and up to two reminders to their respective students who completed the questionnaire as a home-schooling exercise.

Out of *n* = 502 respondents in total, *n* = 364 fully and *n* = 49 partially completed the sorting task. Supplementary Table 1 gives the sample composition by gender, age, education of parents, and completed in the classroom vs. at home.

### 2.2. Procedure and materials

The respondents completed an open card sorting task, assigning 22 climate-relevant behaviours (in card sorting terminology, behaviours refer to cards; the 22 behaviours are listed in Table 1) to four groups (in card sorting terminology, groups refer to piles) in a single sorting routine without repetition. The sorting task was implemented as a drag-and-drop task in the online questionnaire. Respondents were instructed to group those behaviours they regarded as similar and related. Respondents were free to assign as many behaviours to each group as they liked. Respondents were allowed to sort only some of the 22 behaviours and to leave some behaviours unsorted; the following Section “2.3. Analytical approach” describes how missing values from partial completion are addressed in the analysis. The four groups were not named, either by the researchers or by the respondents, and therefore do not carry any meaning apart from indicating that behaviours assigned to the same group are perceived as similar.

The set of 22 behaviours was selected to cover the scope of climate-relevant behaviours that young adults perform in their everyday lives and in which they have agency even while still living in the parental home. Sorting 22 cards is at the upper limit of acceptable respondent burden (Doran et al., 2018). Predefining the number of groups to four avoided the lumper-splitter-problem common in sorting tasks, that is, some respondents lumping cards into very few piles and other respondents splitting cards into very many piles, resulting in high variance in the number of piles (Bernard et al., 2009).

The sorting task was part of a survey study on climate attitudes and behaviours among young adults. Prior to the sorting task, respondents self-reported how frequently they perform the 22 behaviours. It can therefore be assumed that the climate relevance of all behaviours was salient when the respondents started the sorting task. Self-reported behavioural frequency is used to derive the Frequency categorisation (see Section “2.3. Analytical approach” below). Frequency was measured with single items for fifteen behaviours and with multi-item scales for seven behaviours (2−4 items each; aggregated to mean indices; for exact wordings and descriptive statistics see Supplementary Table 2).

Personal norms, stated competencies and environmental knowledge were assessed as potential moderator variables of categorisations. Three personal norms items expressed pro-environmental self-identity and feelings of responsibility and obligation toward climate protection (Steinhorst et al., 2015; Seebauer, 2018). Respondents self-assessed their competencies for engaging in climate-friendly actions with six items on information retrieval, technical skills, and understanding production systems (Nilsson et al., 2017; UNESCO, 2017). Items on personal norms and stated competencies were aggregated to mean indices. Respondents were asked eight quiz questions about effective carbon saving, each quiz question featuring three multiple-choice options with one correct answer (Frick et al., 2004). The quiz questions were aggregated formatively to a sum score of correct answers, ranging from 0 to 8, with higher scores indicating better environmental knowledge. For details of item wordings, response scales, and descriptive statistics, see Supplementary Table 2.

### 2.3. Analytical approach

The unit of analysis is respondents, allowing analysis of respondent attributes as moderator variables. This is in contrast to the exploratory sorting studies by Gabe-Thomas et al. (2016) and Kneebone et al. (2018) who use the piles produced by respondents as a unit of analysis.

The observed similarity pattern (i.e., how each respondent placed behaviours in groups) is compared to five posited categorisations: Domain, Location, Impact, Difficulty, and Frequency. All posited categorisations consist of four categories in order to conform with the group limit in the sorting task and to ensure equal probability of random assignment. The Domain, Location, Impact, and Difficulty categorisations are derived from previous research (see Section “1.3. Categorisations of climate-relevant behaviour”). The Frequency categorisation uses the four factors with the highest eigenvalue in a principal component analysis of self-reported behavioural frequency (see Supplementary Table 3); thus, the Frequency categorisation pools behaviours in the same factor if they are performed with similar frequency.

The analysis adopts a confirmatory rationale and statistically tests how well posited categories fit with the observed responses. *Co-occurrence matrices*, *edit distances*, and *similarity indices* are used to compare categorisations. These three approaches complement each other, as they test either entire categorisations (co-occurrence matrix and edit distance) or specific categories (similarity index), either for the entire sample (co-occurrence matrix) or within each respondent (edit distance and similarity index).

The *co-occurrence matrix* is organised as an item-by-item table with 22 rows and 22 columns. The observed co-occurrence matrix is available for the *n* = 364 subsample who sorted all 22 behaviours and it lists for all pairs of behaviours how many respondents assigned these two behaviours to the same group (Supplementary Table 4). The entries in the matrix cells can range from 0 (no one considers the two behaviours similar) to 364 (all respondents consider the two behaviours similar). The observed distribution in the co-occurrence matrix is compared to several expected distributions: Equal1, the null hypothesis of stochastic independence with the expected frequency in each cell calculated from the row sums and column sums of the observed matrix as in a common Chi^2^ crosstabs test. Equal2, the null hypothesis of equal distribution from random assignment with a 25% probability per group, that is, an expected frequency of 364*0.25 in each cell, except the matrix diagonal with an expected frequency of 364. The distributions as stated by the Domain, Location, Impact, Difficulty, and Frequency categorisations, with an expected frequency of 364*0.95 = 345.8 in each cell where the categorisation posits a pair of behaviours to belong to the same category, an expected frequency of 364*0.05 = 18.2 in all other cells, and an expected frequency of 364 in the matrix diagonal. The 0.95/0.05 multiplicator allows for a 5% error rate by respondents and fulfils the technical requirement of Chi^2^ tests of expected frequencies >5 in each cell. As in a common Chi^2^ crosstabs test, the Chi^2^ test statistic is calculated within each cell as the squared difference between observed and expected frequency divided by the expected frequency, totalled over all cells. With the exception of Equal1, the total Chi^2^ test statistic is halved, because the upper and lower triangle of the matrix along the diagonal are symmetrical. In the same logic of correcting for symmetry, degrees of freedom are reduced by 22 for the cells in the matrix diagonal, which must number 364 both in the observed and the expected matrix, and then halved. Because differences between observed and expected frequencies accumulate over many cells, the Chi^2^ test statistics yield very high numbers that by far exceed critical Chi^2^ values for statistical significance. Still, the Chi^2^ test statistics of the respective categorisations may be compared to assess their relative fit.

The *edit distance* is a combinatorial function that shows how far apart a respondent’s sort is from the posited categorisation. For each respondent, the edit distance gives the minimal number of behaviours they would have to move between groups in order to convert their sort to perfectly represent the posited categorisation. The edit distance is available for the *n* = 364 subsample who sorted all 22 behaviours. This approach recognises that the groups the behaviours are sorted into are not independent of each other. The edit distance is averaged over all respondents and then tested for statistical significance *via* confidence intervals: if the lower bound of the confidence interval does not include 0, the observed similarity pattern deviates significantly from the respective categorisation. If the confidence intervals of the mean edit distances of different categorisations do not overlap, it may be concluded that some categorisations fit better with the observed data than others.

For the *similarity index*, the raw data are recoded into binary variables for each pair of behaviours, coded 1 if these two behaviours are assigned to the (any) same group and coded 0 if they are assigned to (any) different groups. The similarity index is calculated by averaging the binary variables of all pairs of behaviours included in the posited category, resulting in a value between 1 and 0 for each respondent. Its mirror, the dissimilarity index, is the average over the pairwise comparisons between all behaviours not included in the posited category, also resulting in a value between 1 and 0 for each respondent. (Dis-)similarity indices are calculated for each category within each posited categorisation. The indices are available for the *n* = 413 sample who at least partially completed the sorting task because averaging within each category allows correction for missing values. Moreover, averaging allows comparison of categories comprising different numbers of behaviours. Testing for statistical significance is implemented *via* confidence intervals: for the observed similarity pattern to conform to the posited category, the upper bound of the confidence interval of the similarity index should include 1, and the lower bound of the confidence interval of the dissimilarity index should include 0. The lower bound of the confidence interval of the similarity index and the upper bound of the confidence interval of the dissimilarity index should not include 0.25 to reject the null hypothesis that behaviours were sorted randomly. Two categories within the same categorisation can be considered separate concepts if the confidence intervals of their (dis-)similarity indices do not overlap.

Potential *moderator effects* of personal norms, stated competencies and environmental knowledge are assessed by comparing the Chi^2^ test statistics of two co-occurrence matrices obtained by splitting the sample by the median of the respective moderator variable; and by correlating edit distances and similarity indices with the moderator variables.

Confidence intervals use a significance level of *p* < 0.05. Testing *via* confidence intervals is analogue to *t*-tests: for instance, testing whether a confidence interval does not include 0 corresponds to a one-sample *t*-test whether a mean is significantly different from 0; testing whether confidence intervals overlap corresponds to a *t*-test comparing group means. The null hypotheses of an edit distance of 0 or a similarity index of 1 assume perfect congruence with the posited categorisation. These strict tests are likely to be rejected; however, the main interest of the present study does not lie in determining statistical significance, but in comparing which categorisations fit better with the observed similarity patterns than others.

## 3. Results

### 3.1. Co-occurrence matrices

Testing how well entire categorisations fit with the entire sample shows that the respondents do not seem to have a shared mental representation of which climate-relevant behaviours belong together. Equal2 and Equal1, the two null hypotheses assuming random assignment and stochastic independence, have the lowest Chi^2^ test statistics and therefore fit best with the observed data (Table 2). Presumably, the individual patterns of how respondents sorted behaviours to groups differ widely and level each other out, resulting in an overall random pattern in the co-occurrence matrix.

**TABLE 2:** Chi^2^ test statistics of co-occurrence matrices.

	Equal1	Equal2	Domain	Location	Impact	Difficulty	Frequency
All	57,230	24,327	78,081	127,268	88,837	117,970	113,744
Personal norms high	2.31	1.00	3.37	5.17	3.65	4.88	4.64
Personal norms low	2.38	1.00	3.02	5.26	3.65	4.78	4.72
Stated competencies high	2.33	1.00	3.32	5.23	3.70	4.93	4.76
Stated competencies low	2.37	1.00	3.08	5.22	3.59	4.75	4.60
Environmental knowledge high	2.31	1.00	3.40	5.24	3.77	5.05	4.73
Environmental knowledge low	2.38	1.00	3.02	5.20	3.52	4.66	4.62
df	441	210	210	210	210	210	210

All: Chi^2^ values in the *n* = 364 sample. High/low median split subsamples of moderator variables: Chi^2^ values divided by the Equal2 Chi^2^ value to facilitate comparison between different subsample sizes.

However, Chi^2^ test statistics may still be compared to assess which posited categorisations fit better than others. Ranking the Chi^2^ test statistics (Table 2, top row) suggests that the Domain categorisation fits best, followed by Impact, and then with substantially higher Chi^2^ values followed by Frequency, Difficulty, and finally Location.

This ranking of categorisations is not moderated by personal norms, stated competencies, and environmental knowledge. For easier comparison, Table 2 presents the Chi^2^ test statistics as multiples of the Equal2 baseline in the respective subsample. Within each high or low subsample, the ranking of categorisations is the same as in the full sample; for instance, the ranking from Domain to Impact, Frequency, Difficulty, and finally Location applies to high personal norms as well as to low personal norms. Thus, it does not seem to depend on the respondents’ personal norms, stated competencies, and environmental knowledge which categorisation they apply in their similarity patterns.

### 3.2. Edit distances

Comparing the edit distances also tests the fit of entire categorisations, but shows how far individual respondents deviate from the posited categorisation. Mean edit distances range from 7 to 10 (Table 3), meaning respondents would, on average, have to move 7−10 behaviours to another group in order to comply with the posited categorisation. All lower bounds of the confidence intervals are higher than 0, thus according to the strict null hypothesis all categorisations must be rejected (Table 3). Still, this does not preclude comparing the fit between categorisations. The mean edit distances replicate the ranking observed in the co-occurrence matrices, with Domain showing the best fit, followed by Impact, Frequency, Difficulty, and finally Location with increasingly higher edit distances. However, the decrease in fit by edit distance from Impact to Frequency is less pronounced than the decrease in fit by co-occurrence matrices, and the Impact and Frequency edit distances do not differ statistically significantly, since their confidence intervals overlap.

**TABLE 3:** Descriptives, confidence intervals and correlations of edit distances.

	Domain	Location	Impact	Difficulty	Frequency
Mean	7.78	10.63	9.06	9.72	9.15
Standard deviation	2.12	1.57	1.63	1.68	1.83
Lower bound of 95% confidence interval	7.56	10.47	8.89	9.54	8.97
Upper bound of 95% confidence interval	8.00	10.79	9.23	9.89	9.34
Correlation with personal norms	0.01	0.00	−0.05	0.04	0.02
Correlation with stated competencies	0.05	0.00	0.04	0.04	0.10
Correlation with environmental knowledge	−0.01	0.01	0.02	0.05	0.03

*n* = 364.

Again, there is no indication of moderator effects by personal norms, stated competencies, and environmental knowledge. Correlation coefficients between edit distances and moderator variables in Table 3 are *r* < | 0.10| throughout, suggesting that the individual level of personal norms, stated competencies, and environmental knowledge is unrelated to the degree to which a respondent represents a specific categorisation in their similarity pattern.

### 3.3. Similarity indices

Similarity indices provide a nuanced picture of how well respondents conform to specific categories within a categorisation. As above, the strict null hypothesis must be rejected for all categories in all categorisations, because the upper bounds of the confidence intervals of all similarity indices are lower than 1 (Tables 4−8) and the lower bounds of all confidence intervals of all dissimilarity indices are higher than 0 (Supplementary Tables 5−9). The result in the co-occurrence matrices that the Equal2 baseline fits best reappears here in that the upper bounds of the confidence intervals of the dissimilarity indices include 0.25, indicating that respondents may have assigned behaviours to groups randomly. By contrast, the lower bounds of the confidence intervals of the similarity indices all exceed 0.25, pointing to some shared, non-random mental representations held by the respondents. Yet, as above, despite rejecting the null hypothesis the fit of categories may be compared, with the added benefit that similarity indices allow comparison within as well as between categorisations.

**TABLE 4:** Descriptives, confidence intervals and correlations of similarity indices in the Domain categorisation.

	Consumption	Transport	Waste	Advocacy
*n*	409	398	391	389
Mean	0.40	0.51	0.89	0.86
Standard deviation	0.15	0.32	0.31	0.24
Lower bound of 95% confidence interval	0.38	0.48	0.86	0.84
Upper bound of 95% confidence interval	0.41	0.54	0.92	0.88
Correlation with personal norms	0.00	−0.06	0.06	0.00
Correlation with stated competencies	0.03	−0.02	0.06	−0.05
Correlation with environmental knowledge	0.05	−0.07	0.08	−0.01

**TABLE 5:** Descriptives, confidence intervals and correlations of similarity indices in the Location categorisation.

	Indoor	Outdoor	Online	Political space
*n*	406	408	384	377
Mean	0.40	0.32	0.28	0.92
Standard deviation	0.17	0.13	0.12	0.28
Lower bound of 95% confidence interval	0.39	0.31	0.27	0.89
Upper bound of 95% confidence interval	0.42	0.34	0.29	0.94
Correlation with personal norms	−0.01	0.11	0.01	−0.03
Correlation with stated competencies	0.04	0.02	0.02	0.04
Correlation with environmental knowledge	0.05	0.12	0.01	−0.13

**TABLE 6:** Descriptives, confidence intervals and correlations of similarity indices in the Impact categorisation.

	>10%	5−10%	<5%	No direct impact
*n*	383	399	410	389
Mean	0.46	0.31	0.36	0.86
Standard deviation	0.38	0.18	0.13	0.24
Lower bound of 95% confidence interval	0.43	0.29	0.34	0.84
Upper bound of 95% confidence interval	0.50	0.32	0.37	0.88
Correlation with personal norms	0.02	−0.01	0.15	0.00
Correlation with stated competencies	−0.01	−0.01	0.09	−0.05
Correlation with environmental knowledge	0.11	0.00	0.09	−0.01

**TABLE 7:** Descriptives, confidence intervals and correlations of similarity indices in the Difficulty categorisation.

	0−25%	26−50%	51−75%	76−100%
*n*	392	401	395	399
Mean	0.62	0.30	0.33	0.38
Standard deviation	0.19	0.15	0.15	0.20
Lower bound of 95% confidence interval	0.60	0.29	0.31	0.36
Upper bound of 95% confidence interval	0.64	0.32	0.34	0.40
Correlation with personal norms	0.00	−0.03	0.05	−0.03
Correlation with stated competencies	−0.03	−0.02	−0.01	0.05
Correlation with environmental knowledge	−0.01	−0.05	0.03	0.05

**TABLE 8:** Descriptives, confidence intervals and correlations of similarity indices in the Frequency categorisation.

	Factor1	Factor2	Factor3	Factor4
*n*	389	395	396	405
Mean	0.52	0.42	0.31	0.43
Standard deviation	0.17	0.18	0.21	0.16
Lower bound of 95% confidence interval	0.50	0.40	0.29	0.41
Upper bound of 95% confidence interval	0.54	0.43	0.33	0.44
Correlation with personal norms	−0.03	0.09	−0.05	0.05
Correlation with stated competencies	−0.05	0.01	−0.02	0.02
Correlation with environmental knowledge	0.00	0.02	−0.05	0.03

The highest similarity indices emerge in the waste category of the Domain categorisation (*M* = 0.89) on the one hand; and the advocacy category in Domain (*M* = 0.86), political space in Location (*M* = 0.92) no direct impact in Impact (0.86) and high Difficulty (0−25%: *M* = 0.62) on the other hand. Advocacy, political space, no direct impact and high difficulty reach similarly high indices because they all include selected behaviours of political and civil engagement (behaviours 18−22, see Table 1). These categories seem to reflect shared representations of related behaviours that most respondents hold.

The following have low similarity indices: the energy use and consumption category in the Domain categorisation (*M* = 0.40); indoor, outdoor, and online in Location (*M* = 0.40, *M* = 0.32, *M* = 0.28, respectively); the middle categories in Impact (5−10%: *M* = 0.31; <5%: *M* = 0.36); the middle categories in Difficulty (26−50%: *M* = 0.30; 51−75%: *M* = 0.33); and Factor3 in Frequency (*M* = 0.31). These all indicate that respondents hardly agree that the behaviours within these categories belong together. The umbrella character of energy use and consumption comprising half of all investigated behaviours might make it likely that respondents assign some of these many behaviours to another group. Distinctions between categories could be more blurry in the middle than at the extremes of the Impact and Difficulty spectrum. Location just does not seem to be a mental concept the respondents use to structure their climate-relevant activities.

However, despite the overall low means in similarity indices, hardly any confidence intervals overlap between categories of the same categorisation. This indicates that some categories represent perceived similarity better than others do. For instance, even in Location, the categorisation least adopted by the respondents, it may still be concluded that the indoor category (*M* = 0.40) reflects significantly better lay mental representations than do the outdoor or online categories (*M* = 0.32, *M* = 0.28).

Interestingly, the high Impact (>10% share of the personal carbon footprint: *M* = 0.46) and the high Difficulty (0−25% engagement probability: *M* = 0.62) categories stand out against the other categories of Impact and Difficulty. Behaviours with a high carbon footprint (heating, flying, and eating meat) and behaviours that are performed by few other people (buying organic food, various political, and civil engagement behaviours) seem to be perceived as separate from less carbon-intensive and less difficult behaviours. That high impact and high difficulty behaviours go together is also reflected in the *M* = 0.52 similarity index of Factor1 in Frequency (eating meat, various political and civil engagement behaviours).

Consistent with the results in co-occurrence matrices and edit distances, the moderator variables personal norms, stated competencies, and environmental knowledge do not have any discernible effect on similarity indices. Correlations of *r* < | 0.15| in all categorisations (Tables 4−8) indicate that even the perceived similarity within specific categories does not depend on the norms, competencies, or knowledge a respondent has.

## 4. Discussion and conclusion

The present paper compares the perceived similarity of climate-relevant behaviours to five posited categorisations: Domain, Location, Impact, Difficulty, and Frequency. Co-occurrence matrices, edit distances and similarity indices assess in a confirmatory approach how well the posited categorisations fit with the similarity patterns observed in an open card sorting task.

The best fit is found for the null hypotheses of stochastic independence and random assignment, suggesting there is no common structure to the respondents’ sorting and thus no shared mental representation of similarity held by all respondents. This resonates with Gabe-Thomas et al.’s (2016) finding of substantial disagreement between laypeople in how they categorise behaviours. None of the posited categorisations can be statistically confirmed. Personal norms, stated competencies, and environmental knowledge do not influence perceived similarity. This puts into question the validity of categorisations used by experts and found in exploratory sorting studies. However, this overall result should not be mistaken to discard previous research, as the statistical testing uses strict null hypotheses that are likely to be rejected. Instead, the present study should be taken as a starting point that indicates which categorisations and categories feature higher or lower perceived similarity.

Indeed, some categories of behaviours perceived as more similar than others emerge. Organising behaviours in a Domain taxonomy seems to relatively best represent how young adults mentally structure their everyday actions. The Impact categorisation performs second best. The Difficulty and Frequency categorisations can be differentiated, as indicated by the difference in Chi^2^ test statistics and edit distance; this contradicts Truelove and Gillis, 2018 finding that laypeople tend to confound these two categorisations. Throughout, categories within the same categorisations are perceived as distinct, as indicated by differences in similarity indices; presumably, these categories do structure, albeit weakly, how young adults think about climate-relevant behaviours.

Within the Domain categorisation, waste behaviours (behaviours 7 and 8, separating waste and avoiding plastic) and advocacy behaviours (behaviours 18−22, participating in an non-governmental organisation (NGO) for climate protection, speaking out for climate protection online, speaking with other people about climate protection, donating for climate protection, demonstrating for climate protection) belong together in laypeople’s mental representations. Behaviours of high Impact (behaviours 1, 12, and 15, heating, flying, and eating meat) and high Difficulty (behaviours 16 and 18−22, buying organic food and the above advocacy behaviours) seem to stand out and form a subset of similar meaning within lay mental representations. Future research could follow up on this interesting finding and explore how behaviours at the extreme end of the spectrum relate to and possibly advance each other.

### 4.1. Implications for policy

Perceived similarity may provide orientation on which behaviours should be promoted in concert in order to communicate comprehensibly and to catalyse spillover (see Sections “1.2. Catalysing spillover by means of perceived similarity” and “1.3. Categorisations of climate-relevant behaviour”). Presumably, consumers are more likely to listen to and adopt persuasive arguments that refer to behaviours they perceive as similar.

The results suggest designing policy interventions for broad behavioural change around Domain categories, as this categorisation has the relatively best fit with the observed data. Promoting selected behaviours from the waste or advocacy categories is likely to carry over to other behaviours within the same category. By contrast, campaigns focussing on Location, the worst-fitting categorisation, are likely to fall short; for instance, promoting various outdoor behaviours together could not be expected to catalyse spillover.

If a campaign is bound to a specific categorisation, it should focus on the best-performing category within this categorisation. Take the example of an intervention using descriptive social norms, in other words, the effect that the actions observed in important others serve as a cue for socially accepted behaviours (Staats et al., 2004). This intervention would communicate to its audience how many other people already perform the target behaviours, which equals engagement probability in the Difficulty categorisation. Difficulty has weak overall fit, as shown by the co-occurrence matrix and the edit distance, but its categories clearly differ in the similarity indices. When communicating single behaviours, behaviours from the 76−100% Difficulty category could be highlighted (e.g., turning off the lights, saving hot water), because large parts of the population already perform these behaviours and therefore convey a strong descriptive norm. When communicating bundles of behaviours, it could be recommended to address 0−25% Difficulty behaviours (e.g., buying organic food, demonstrating for climate protection) because of the substantially higher similarity index of this category. However, since only a few people already perform 0−25% Difficulty behaviours, the intervention would have to highlight selected frontrunner groups as the important others who convey a descriptive norm.

### 4.2. Limitations and directions for future research

As in any other empirical study, the results underlie important caveats which at the same time point to avenues for future research. First and foremost, the findings only apply to young adults in Austria and need to be replicated in other population segments and in other geographies. Sampling young adults rather than the general population might have biassed the results. Young adults can be expected to perceive less similarity between climate-relevant behaviours than grown-ups: They perform pro-environmental behaviours less frequently than grown-ups (Krettenauer, 2017; Wang et al., 2021) and might thus be less likely to experience communalities between behaviours. They still undergo socialisation processes (Haustein et al., 2009; Klöckner and Matthies, 2012) whereas grown-ups already hold common norms that indicate which behaviours are related to each other. Presumably, the present study reports a lower level of perceived similarity than could be expected in the general population. However, entering young adulthood coincides with recovering from an “adolescent dip” in pro-environmental behaviours (Olsson and Gericke, 2016; Keith et al., 2021). Thus, young adults might be about to return to general population levels of perceived similarity. Future research could provide more clarity on how perceived similarity shifts over the life course, either in longitudinal studies or by comparing age cohorts. The present study sample is quite homogeneous in terms of age, educational level and living situation; this homogeneity presumably works in favour of shared mental representations. Thus, future studies using more diverse samples might find an even worse fit of the categorisations analysed here.

Similarity indices are biassed to indicate lower similarity in categories with a higher number of posited behaviours because consistency is harder to achieve in categories comprising many behaviours, as the pairwise binaries may level each other out and may converge to a within-respondent average of 0.5. This bias could, for instance, explain why in the Domain categorisation the waste category (two behaviours, *M* = 0.89) scores a higher similarity index than the energy use and consumption category (11 behaviours, *M* = 0.40). Thus, future studies could be advised to compare categories comprising an equal number of behaviours.

Another limitation to be noted is that the assignment of behaviours to categories in each of the Domain, Location, Impact, Difficulty, and Frequency categorisations is not clear-cut. All five categorisations involve some ambiguities in the posited assignment of behaviours to categories. In Domain, energy use and consumption is an umbrella category comprising highly diverse behaviours. In Location, buying clothes and electronic devices (behaviours 13 and 14) are assigned to the online location because these two product segments have the largest share in Austrian online commerce (Handelsverband, 2022), presumably even more so among the young adult population studied here; however, clothes and electronic devices may also be bought outdoors in brick-and-mortar stores. In Location, avoiding plastic (behaviour 8) starts indoor at home when packing refillable containers but is realised outdoor when buying unpackaged food. Location could alternatively use places such as home, job/school, or free time activities as categories. In Impact, the shares of behaviours in the carbon footprint show substantial interpersonal variation, and donating (behaviour 21) may have a direct impact when buying carbon emission certificates. In Difficulty, engagement probabilities vary considerably between the Swiss, Dutch, and German estimates produced by Kaiser and colleagues, and their attitude distributions might be outdated since they were calculated in the 2000s. In Frequency, some behaviours have cross-loadings on other factors (e.g., behaviour 15, eating meat). Error from these ambiguities might reduce the statistical fit of posited categorisations. Future studies could attempt to clarify these ambiguities in an exploratory pre-study; or could systematically vary and compare to which category the respective behaviours are assigned, which however brings the risk of overfitting to the data and shifting from the confirmatory to a more exploratory approach.

Finally, due to constraints in survey administration, the respondents were not asked to name the groups of behaviours they formed in the card sorting task. Naming groups is common practice in exploratory sorting studies (e.g., Gabe-Thomas et al., 2016). Future research should elicit the subjective meanings of the categories formed by respondents and could check to which extent the group names mirror the names of posited categories.

Still, the present paper introduces three analytical approaches for confirmatory testing of posited categorisations against observed similarity patterns: co-occurrence matrix, edit distance, and similarity index. After valuable exploratory studies in recent years (Gabe-Thomas et al., 2016; Doran et al., 2018; Kneebone et al., 2018), these approaches may help in consolidating research–not just on the mental representations of climate-relevant behaviours, but also on cognitive biases and misleading beliefs among climate sceptics (Zhao and Luo, 2021), or potentially for any card sorting data.

## Data availability statement

The raw data supporting the conclusions of this article will be made available by the authors, without undue reservation.

## Ethics statement

Ethical review and approval was not required for the study on human participants in accordance with the local legislation and institutional requirements. Written informed consent from the participants’ legal guardian/next of kin was not required to participate in this study in accordance with the national legislation and the institutional requirements.

## Author contributions

SS and HE: methodology and writing—review and editing. Both authors contributed to the article and approved the submitted version.
