# Supplementary material for: Mental representation of climate-relevant behaviours: Confirmatory testing of similarity patterns obtained in a card sorting task by young adults
Source: Front Psychol. 2023 Feb 9;14:1117452. doi: 10.3389/fpsyg.2023.1117452 (PMC9947702; doi:10.3389/fpsyg.2023.1117452)
Supplement: Supplementary file 1 [file Data_Sheet_1.docx]

Supplementary Material

Mental representation of climate-relevant behaviours: Confirmatory testing of similarity patterns obtained in a card sorting task

Sebastian Seebauer^*^, Hans Peter Ellmer

*** Correspondence: s**ebastian.seebauer@joanneum.at

Table S.1. Sample composition.

|  | **Female** | **Age (years)** | | | | | |  | | **Education of parents** | | | | |  |
| --- | --- | --- | --- | --- | --- | --- | --- | --- | --- | --- | --- | --- | --- | --- | --- |
|  |  | 16 | 17 | 18 | 19 | 20 | 21 | |  | | Comp. | Voc. | Sec. | High | |
| N=413  partially or fully completed sorting task | 49.0 | 0.3 | 27.6 | 44.5 | 24.3 | 3.0 | 0.5 | |  | | 4.0 | 24.1 | 30.2 | 41.7 | |
| N=300  completed in classroom | 42.6 | 0.3 | 28.3 | 40.5 | 26.4 | 3.7 | 0.7 | |  | | 4.6 | 24.7 | 30.7 | 40.0 | |
| N=113  completed at home | 67.3 | 0.0 | 25.0 | 55.8 | 18.3 | 1.0 | 0.0 | |  | | 2.1 | 22.4 | 28.7 | 46.8 | |
| N=364 fully completed sorting task | 50.0 | 0.0 | 28.1 | 44.3 | 24.4 | 3.1 | 0.0 | |  | | 4.0 | 24.0 | 29.9 | 42.1 | |
| N=262  completed in classroom | 42.2 | 0.0 | 29.9 | 39.1 | 27.1 | 3.9 | 0.0 | |  | | 4.5 | 25.4 | 29.9 | 40.2 | |
| N=102  completed at home | 71.3 | 0.0 | 23.4 | 58.5 | 17.0 | 1.1 | 0.0 | |  | | 2.4 | 20.3 | 29.7 | 47.6 | |

Table gives relative frequencies in percentages. Education of parents: highest educational level attained by either parent. Comp. = Compulsory education; Voc. = Vocational education; Sec. = Secondary school (with school-leaving exam); High = Higher education (university-level).

Table S.2. Descriptives and response scales of behaviour and moderator items.

| **Factor** | **Item** | **Response scale** | **N** | **Mean** | **SD** | **Cronbach’s α** |
| --- | --- | --- | --- | --- | --- | --- |
| Personal norms | If I had to describe myself, my mindset about climate protection would play a role. | 1=fully disagree to 5=fully agree | 411 | 2.91 | 1.11 | .81 |
|  | I see it as my responsibility to contribute to climate protection. |  | 411 | 3.69 | 1.12 |  |
|  | I see myself as a person who considers climate protection. |  | 407 | 3.69 | 1.07 |  |
| Stated competencies | How good are you at… Researching and assessing information | -2=very bad to +2=very good | 407 | 1.18 | 0.80 | .63 |
|  | Understanding how technical devices work |  | 407 | 1.00 | 1.06 |  |
|  | Calculating your personal ecological footprint |  | 407 | 0.04 | 1.26 |  |
|  | Comparing products and providers |  | 407 | 0.89 | 0.93 |  |
|  | Employing different approaches to get answers to your questions |  | 407 | 0.93 | 0.84 |  |
|  | Understanding the consequences of different production processes and consumption patterns |  | 407 | 0.67 | 0.98 |  |
| Environmental knowledge | Which household domain consumes the most energy? a) **heating** b) lighting c) water heating) | 1=correct to 0=incorrect | 405 | 0.56 | 0.50 | - |
|  | What consumes the least energy when heating water? a) **Electric kettle** b) Electric stove c) Gas stove |  | 405 | 0.56 | 0.50 |  |
|  | What percentage of reduction in CO2 emission can be achieved through a plastic bag ban? a) 1% b) 4% c) **0.01 %** |  | 405 | 0.19 | 0.39 |  |
|  | Which of the following materials is best suited for recycling? a) **Aluminium** b) Plastic bottles c) Green glass |  | 405 | 0.16 | 0.37 |  |
|  | What is the most energy-efficient way to ventilate in winter? a) **Leave windows open for up to 10 minutes** b) Leave the window open for more than 10 minutes c) Leave windows tilted for more than 10 minutes |  | 405 | 0.87 | 0.33 |  |
|  | The carbon emissions of a 100 km car journey correspond to a) 12 km train ride b) 120 km train ride c) **1,200 km train ride**. |  | 405 | 0.66 | 0.47 |  |
|  | How much lower is the carbon footprint of organic milk compared to conventional milk? a) 10% b) **30%** c) 70% |  | 405 | 0.66 | 0.47 |  |
|  | Which type of meat has the largest CO_2_ footprint? a) **Beef** b) Pork c) Chicken |  | 405 | 0.75 | 0.43 |  |
| 1. Heating | Put on warm clothing rather than turn on the heating if the room is cold | 1=never to 5=always | 501 | 4.05 | 1.13 |  |
| 1. Showering | Frequency of showering in a typical week | Days per week | 501 | 5.80 | 2.24 | - |
| 1. Saving hot water | Take shorter showers to save water | 1=never to 5=always | 501 | 2.81 | 1.18 | - |
| 1. Turning on the light | Turn off the lights when not needed | 1=never to 5=always | 502 | 4.49 | 0.75 | - |
| 1. Using electronic devices | Disconnect electronic devices from the mains when not using them | 1=never to 5=always | 501 | 2.97 | 1.30 | - |
| 1. Streaming video | Frequency of video streaming (such as Netflix, YouTube, Amazon Prime, Sky) on a typical day | Hours per day | 501 | 2.39 | 1.60 | - |
| 1. Separating waste | Not throw anything in the residual waste for which there are separate bins | 1=never to 5=always | 502 | 4.01 | 1.12 | - |
| 1. Avoiding plastic | Buy products in reusable packaging (e.g. returnable bottles) | 1=never to 5=always | 502 | 3.42 | 0.83 | .61 |
|  | Avoid plastic packaging |  | 501 | 3.23 | 0.86 |  |
| 1. Using the car | Modal split of car on trips for education (e.g. school, side job, private lessons) | Percentage of trips of the respective trip type | 483 | 34.1 | 37.9 | .86 |
|  | for errands (e.g. supermarket, doctor, bringing or picking up someone) |  | 469 | 60.9 | 40.5 |  |
|  | for hobbies (e.g. sports, association, music school) |  | 408 | 54.6 | 42.4 |  |
|  | for leisure (e.g. activities with friends, clubbing, events, concerts) |  | 471 | 55.8 | 41.0 |  |
| 1. Using public transport | Modal split of public transport on trips for education (e.g. school, side job, private lessons) | Percentage of trips of the respective trip type | 483 | 45.9 | 38.1 | .76 |
|  | for errands (e.g. supermarket, doctor, bringing or picking up someone) |  | 469 | 11.9 | 23.2 |  |
|  | for hobbies (e.g. sports, association, music school) |  | 407 | 17.8 | 30.1 |  |
|  | for leisure (e.g. activities with friends, clubbing, events, concerts) |  | 471 | 27.8 | 33.2 |  |
| 1. Using the bicycle | Modal split of cycling on trips for education (e.g. school, side job, private lessons) | Percentage of trips of the respective trip type | 483 | 4.7 | 16.3 | .85 |
|  | for errands (e.g. supermarket, doctor, bringing or picking up someone) |  | 469 | 6.7 | 18.6 |  |
|  | for hobbies (e.g. sports, association, music school) |  | 407 | 8.7 | 22.0 |  |
|  | for leisure (e.g. activities with friends, clubbing, events, concerts) |  | 471 | 3.8 | 12.9 |  |
| 1. Flying | Number of flights in the last year | Number | 501 | 1.09 | 1.45 | - |
| 1. Buying clothes | Buy new clothes and shoes or get them as a present to be always dressed fashionably | 1=never to 5=always | 502 | 2.94 | 1.05 | - |
| 1. Buying electronic devices | Buy new electronic devices or get them as a present to always have the best available technology | 1=never to 5=always | 501 | 2.41 | 1.04 | - |
| 1. Eating meat | Eat meat products | 1=never to 5=always | 502 | 3.42 | 1.26 | - |
| 1. Buying organic food | Buy organic food products | 1=never to 5=always | 501 | 3.45 | 1.07 | - |
| 1. Buying local food | Buy food products produced in Austria | 1=never to 5=always | 501 | 3.83 | 0.69 | - |
| 1. Participating in an NGO for climate protection | Be a member of an NGO that campaigns for climate protection | 1=yes to 0=no | 501 | 0.01 | 0.12 | .53 |
|  | Like webpages of NGOs that campaign for climate protection |  | 501 | 0.32 | 0.47 |  |
|  | Share online content of NGOs that campaign for climate protection |  | 501 | 0.12 | 0.33 |  |
|  | Distribute information materials of NGOs that campaign for climate protection |  | 501 | 0.02 | 0.14 |  |
| 1. Speaking out for climate protection online | Participate in online discussions on climate protection | 1=yes to 0=no | 501 | 0.16 | 0.36 | .64 |
|  | Convince others online of the importance of climate protection |  | 501 | 0.20 | 0.40 |  |
|  | Comment on online postings on climate protection |  | 501 | 0.12 | 0.32 |  |
| 1. Speaking with other people about climate protection | Convince others in personal conversations of the importance of climate protection | 1=yes to 0=no | 501 | 0.62 | 0.49 | .70 |
|  | Debate in personal conversations about climate protection |  | 501 | 0.72 | 0.45 |  |
| 1. Donating for climate protection | Donate for climate protection | 1=yes to 0=no | 501 | 0.07 | 0.26 |  |
| 1. Demonstrating for climate protection | Participate in demonstrations or protests for climate protection | 1=yes to 0=no | 501 | 0.27 | 0.44 |  |

N=valid responses. SD=Standard deviation. Correct answers in quiz questions printed in bold.

Table S.3. Principal component analysis by behavioural frequency.

| **Item** | **Factor1** | **Factor2** | **Factor3** | **Factor4** |
| --- | --- | --- | --- | --- |
| 1. Heating |  | **.45** |  | .28 |
| 1. Showering |  | -.27 |  | **.44** |
| 1. Saving hot water | .28 | **.48** |  | .26 |
| 1. Turning on the light |  | **.50** |  |  |
| 1. Using electronic devices |  | **.51** |  |  |
| 1. Streaming video |  |  |  | **-.58** |
| 1. Separating waste |  |  |  | **.29** |
| 1. Avoiding plastic | .43 |  |  | **.53** |
| 1. Using the car |  |  | **-.88** |  |
| 1. Using public transport |  |  | **.73** |  |
| 1. Using the bicycle |  |  | **.43** |  |
| 1. Flying |  | **-.47** |  |  |
| 1. Buying clothes |  | **-.59** | -.31 |  |
| 1. Buying electronic devices |  | **-.68** |  |  |
| 1. Eating meat | **-.38** |  |  | -.30 |
| 1. Buying organic food | .42 |  |  | **.56** |
| 1. Buying local food |  |  |  | **.64** |
| 1. Participating in an NGO for climate protection | **.69** |  |  |  |
| 1. Speaking out for climate protection online | **.69** |  |  |  |
| 1. Speaking with other people about climate protection | **.57** |  |  |  |
| 1. Donating for climate protection | **.50** |  |  |  |
| 1. Demonstrating for climate protection | .41 |  | **.52** |  |
| Eigenvalue | 4.02 | 1.93 | 1.76 | 1.33 |
| Explained variance | 18.3% | 8.8% | 8.0% | 6.1% |

N=502. Varimax rotation. Rotated factor loadings with loadings <.20 omitted. Factor loadings are printed in bold for the factor the behaviour is assigned to.

Table S.4. Observed co-occurrence matrix.

| **Item** | **1** | **2** | **3** | **4** | **5** | **6** | **7** | **8** | **9** | **10** | **11** | **12** | **13** | **14** | **15** | **16** | **17** | **18** | **19** | **20** | **21** | **22** |
| --- | --- | --- | --- | --- | --- | --- | --- | --- | --- | --- | --- | --- | --- | --- | --- | --- | --- | --- | --- | --- | --- | --- |
| 1. Heating | 364 | 262 | 99 | 289 | 227 | 223 | 63 | 47 | 165 | 20 | 28 | 139 | 193 | 183 | 187 | 20 | 20 | 3 | 5 | 3 | 6 | 5 |
| 1. Showering | 262 | 364 | 149 | 256 | 201 | 193 | 108 | 91 | 106 | 58 | 67 | 87 | 173 | 154 | 149 | 61 | 56 | 10 | 10 | 16 | 13 | 13 |
| 1. Saving hot water | 99 | 149 | 364 | 94 | 60 | 60 | 287 | 270 | 12 | 217 | 210 | 12 | 30 | 42 | 31 | 222 | 220 | 47 | 43 | 62 | 58 | 52 |
| 1. Turning on the light | 289 | 256 | 94 | 364 | 251 | 251 | 62 | 48 | 140 | 24 | 26 | 124 | 195 | 203 | 177 | 20 | 19 | 6 | 9 | 10 | 9 | 8 |
| 1. Using electronic devices | 227 | 201 | 60 | 251 | 364 | 279 | 46 | 34 | 135 | 12 | 20 | 114 | 216 | 268 | 158 | 31 | 30 | 4 | 11 | 8 | 7 | 8 |
| 1. Streaming video | 223 | 193 | 60 | 251 | 279 | 364 | 40 | 31 | 146 | 17 | 23 | 146 | 186 | 238 | 170 | 19 | 18 | 10 | 15 | 14 | 11 | 8 |
| 1. Separating waste | 63 | 108 | 287 | 62 | 46 | 40 | 364 | 325 | 15 | 218 | 212 | 9 | 51 | 39 | 62 | 266 | 262 | 47 | 47 | 67 | 59 | 52 |
| 1. Avoiding plastic | 47 | 91 | 270 | 48 | 34 | 31 | 325 | 364 | 11 | 217 | 208 | 12 | 63 | 39 | 76 | 281 | 283 | 49 | 44 | 65 | 59 | 53 |
| 1. Using the car | 165 | 106 | 12 | 140 | 135 | 146 | 15 | 11 | 364 | 104 | 120 | 323 | 134 | 138 | 181 | 5 | 5 | 1 | 3 | 1 | 3 | 4 |
| 1. Using public transport | 20 | 58 | 217 | 24 | 12 | 17 | 218 | 217 | 104 | 364 | 317 | 102 | 15 | 15 | 12 | 199 | 200 | 49 | 41 | 64 | 60 | 55 |
| 1. Using the bicycle | 28 | 67 | 210 | 26 | 20 | 23 | 212 | 208 | 120 | 317 | 364 | 115 | 20 | 18 | 17 | 189 | 188 | 47 | 38 | 52 | 55 | 51 |
| 1. Flying | 139 | 87 | 12 | 124 | 114 | 146 | 9 | 12 | 323 | 102 | 115 | 364 | 121 | 132 | 181 | 6 | 8 | 4 | 4 | 4 | 5 | 5 |
| 1. Buying clothes | 193 | 173 | 30 | 195 | 216 | 186 | 51 | 63 | 134 | 15 | 20 | 121 | 364 | 251 | 211 | 86 | 87 | 10 | 8 | 11 | 9 | 10 |
| 1. Buying electronic devices | 183 | 154 | 42 | 203 | 268 | 238 | 39 | 39 | 138 | 15 | 18 | 132 | 251 | 364 | 166 | 59 | 57 | 2 | 6 | 3 | 6 | 5 |
| 1. Eating meat | 187 | 149 | 31 | 177 | 158 | 170 | 62 | 76 | 181 | 12 | 17 | 181 | 211 | 166 | 364 | 96 | 93 | 5 | 7 | 6 | 3 | 3 |
| 1. Buying organic food | 20 | 61 | 222 | 20 | 31 | 19 | 266 | 281 | 5 | 199 | 189 | 6 | 86 | 59 | 96 | 364 | 330 | 50 | 43 | 62 | 61 | 51 |
| 1. Buying local food | 20 | 56 | 220 | 19 | 30 | 18 | 262 | 283 | 5 | 200 | 188 | 8 | 87 | 57 | 93 | 330 | 364 | 51 | 45 | 66 | 63 | 51 |
| 1. Participating in an NGO for climate protection | 3 | 10 | 47 | 6 | 4 | 10 | 47 | 49 | 1 | 49 | 47 | 4 | 10 | 2 | 5 | 50 | 51 | 364 | 321 | 303 | 326 | 334 |
| 1. Speaking out for climate protection online | 5 | 10 | 43 | 9 | 11 | 15 | 47 | 44 | 3 | 41 | 38 | 4 | 8 | 6 | 7 | 43 | 45 | 321 | 364 | 328 | 301 | 318 |
| 1. Speaking with other people about climate protection | 3 | 16 | 62 | 10 | 8 | 14 | 67 | 65 | 1 | 64 | 52 | 4 | 11 | 3 | 6 | 62 | 66 | 303 | 328 | 364 | 295 | 307 |
| 1. Donating for climate protection | 6 | 13 | 58 | 9 | 7 | 11 | 59 | 59 | 3 | 60 | 55 | 5 | 9 | 6 | 3 | 61 | 63 | 326 | 301 | 295 | 364 | 320 |
| 1. Demonstrating for climate protection | 5 | 13 | 52 | 8 | 8 | 8 | 52 | 53 | 4 | 55 | 51 | 5 | 10 | 5 | 3 | 51 | 51 | 334 | 318 | 307 | 320 | 364 |

Table S.5. Descriptives, confidence intervals and correlations of dissimilarity indices in the Domain categorisation.

|  | **Consumption** | **Transport** | **Waste** | **Advocacy** |
| --- | --- | --- | --- | --- |
| n | 410 | 412 | 413 | 413 |
| Mean | 0,32 | 0,27 | 0,25 | 0,33 |
| Standard deviation | 0,12 | 0,08 | 0,07 | 0,11 |
| Lower bound of 95% confidence interval | 0,31 | 0,27 | 0,24 | 0,32 |
| Upper bound of 95% confidence interval | 0,34 | 0,28 | 0,26 | 0,34 |

Table S.6. Descriptives, confidence intervals and correlations of dissimilarity indices in the Location categorisation.

|  | **Indoor** | **Outdoor** | **Online** | **Political space** |
| --- | --- | --- | --- | --- |
| n | 410 | 410 | 413 | 413 |
| Mean | 0,26 | 0,29 | 0,26 | 0,26 |
| Standard deviation | 0,08 | 0,10 | 0,08 | 0,08 |
| Lower bound of 95% confidence interval | 0,25 | 0,28 | 0,25 | 0,26 |
| Upper bound of 95% confidence interval | 0,26 | 0,30 | 0,27 | 0,27 |

Table S.7. Descriptives, confidence intervals and correlations of dissimilarity indices in the Impact categorisation.

|  | **>10%** | **5-10%** | **<5%** | **no direct impact** |
| --- | --- | --- | --- | --- |
| n | 413 | 413 | 410 | 413 |
| Mean | 0,26 | 0,25 | 0,27 | 0,33 |
| Standard deviation | 0,08 | 0,08 | 0,09 | 0,11 |
| Lower bound of 95% confidence interval | 0,25 | 0,25 | 0,26 | 0,32 |
| Upper bound of 95% confidence interval | 0,27 | 0,26 | 0,28 | 0,34 |

Table S.8. Descriptives, confidence intervals and correlations of dissimilarity indices in the Difficulty categorisation.

|  | **0-25%** | **26-50%** | **51-75%** | **76-100%** |
| --- | --- | --- | --- | --- |
| n | 413 | 412 | 413 | 412 |
| Mean | 0,33 | 0,26 | 0,25 | 0,24 |
| Standard deviation | 0,11 | 0,08 | 0,08 | 0,07 |
| Lower bound of 95% confidence interval | 0,32 | 0,26 | 0,24 | 0,24 |
| Upper bound of 95% confidence interval | 0,34 | 0,27 | 0,25 | 0,25 |

Table S.9. Descriptives, confidence intervals and correlations of dissimilarity indices in the Frequency categorisation.

|  | **Factor1** | **Factor2** | **Factor3** | **Factor4** |
| --- | --- | --- | --- | --- |
| n | 413 | 412 | 412 | 410 |
| Mean | 0.29 | 0.27 | 0.27 | 0.25 |
| Standard deviation | 0.09 | 0.09 | 0.08 | 0.07 |
| Lower bound of 95% confidence interval | 0.29 | 0.26 | 0.26 | 0.24 |
| Upper bound of 95% confidence interval | 0.30 | 0.28 | 0.27 | 0.26 |
